# Normal Proliferation and Tumorigenesis but Impaired Pancreatic Function in Mice Lacking the Cell Cycle Regulator Sei1

**DOI:** 10.1371/journal.pone.0008744

**Published:** 2010-01-18

**Authors:** Pablo J. Fernandez-Marcos, Cristina Pantoja, Agueda Gonzalez-Rodriguez, Nicholas Martin, Juana M. Flores, Angela M. Valverde, Eiji Hara, Manuel Serrano

**Affiliations:** 1 Tumor Suppression Group, Spanish National Cancer Research Centre (CNIO), Madrid, Spain; 2 Institute of Biomedicine Alberto Sols (CSIC/UAM), Madrid, Spain; 3 Centro de Investigación Biomédica en Red de Diabetes y Enfermedades Metabólicas Asociadas (CIBERDEM), ISCIII, Madrid, Spain; 4 Cancer Institute of the Japanese Foundation for Cancer Research, Tokyo, Japan; 5 Department of Animal Surgery and Medicine, Complutense University of Madrid, Madrid, Spain; University of Bremen, Germany

## Abstract

Sei1 is a positive regulator of proliferation that promotes the assembly of Cdk4-cyclin D complexes and enhances the transcriptional activity of E2f1. The potential oncogenic role of Sei1 is further suggested by its overexpression in various types of human cancers. To study the role of Sei1, we have generated a mouse line deficient for this gene. Sei1-null fibroblasts did not show abnormalities regarding proliferation or susceptibility to neoplastic transformation, nor did we observe defects on Cdk4 complexes or E2f activity. Sei1-null mice were viable, did not present overt pathologies, had a normal lifespan, and had a normal susceptibility to spontaneous and chemically-induced cancer. Pancreatic insulin-producing cells are known to be particularly sensitive to Cdk4-cyclin D and E2f activities, and we have observed that Sei1 is highly expressed in pancreatic islets compared to other tissues. Interestingly, Sei1-null mice present lower number of islets, decreased β-cell area, impaired insulin secretion, and glucose intolerance. These defects were associated to nuclear accumulation of the cell-cycle inhibitors p21^Cip1^ and p27^Kip1^ in islet cells. We conclude that Sei1 plays an important role in pancreatic β-cells, which supports a functional link between Sei1 and the core cell cycle regulators specifically in the context of the pancreas.

## Introduction

In mammalian cells, entry from quiescence into the cell cycle and advance through G1 into S phase are controlled by the activity of the D-type cyclin-dependent kinases Cdk4 and Cdk6 (Cdk4,6/D complexes) [1]. The expression of D-type cyclins in response to extracellular mitogenic signals is tightly regulated at different levels, as well as their binding to Cdk4,6. An important level of regulation is exerted by the Cdk inhibitors (CKIs), which include the Ink4 family (p16^Ink4a^, p15^Ink4b^, p18^Ink4c^ and p19^Ink4d^) and the Cip/Kip family (p21^Cip1^, p27^Kip1^ and p57^Kip2^) [2], [3]. Once active, Cdk4,6/D complexes phosphorylate members of the pocket protein family, namely, Rb, p107 and p130. These proteins bind and inhibit transcription factors important for cell cycle progression, most notably the E2f family of transcription factors, whose target genes are necessary for the replication of the DNA during S-phase. Upon phosphorylation, the pocket proteins are released from the E2f transcription factors allowing them to become transcriptionally active [4]. Alterations in the proteins that regulate the early stages of the cell cycle, including Cdk4, D-type cyclins and E2f1, have proven to be important in the development of cancer [1]. At a more physiological level, Cdk4, D-type cyclins and the E2f family have a clear impact in pancreas development and homeostasis. Mice deficient for these proteins display pancreatic abnormalities, mainly characterized by decreased numbers of β-cells which result in insulin resistance and diabetes [5]-[7]. Together, these observations indicate that β-cells are highly sensitive to the activity of the Cdk4/cyclinD/E2f pathway [8].

Sei1, also known as Sertad1, belongs to the Sertad family of proteins composed by four members named Sertad1-4 [9]. Sei1 binds to and stabilizes Cdk4/D complexes, stimulating their kinase activity and preventing their inhibition by p16^Ink4a^ [10], [11]. Of note, CKIs p21^Cip1^ and p27^Kip1^ also participate in the assembly and stabilization of Cdk4/D complexes, as well as, in their nuclear translocation [12], [13]. However, it is still under debate whether the Cdk4/D complexes associated to p21^Cip1^ or p27^Kip1^ are inactive or may retain activity under particular conditions (discussed in [14]). Independent studies on Sei1 and its closest homologue Sertad2 have indicated that these proteins also promote proliferation by binding to the transcription factor E2f1 and by enhancing its transcriptional activity [15]. Together, these studies suggest that Sei1 plays a positive role in cell cycle progression.

Based on the positive role of Sei1 on Cdk4/D and E2f1 activities, it is conceivable that Sei1 could have a role in cancer and/or pancreatic islet biology. With regard to cancer, amplification of the genomic locus of Sei1 has been found in human head and neck squamous cell carcinomas, ovarian carcinomas and gastric carcinoma [16]–[18]. Here we have tested the current models on Sei1 function by generating and analyzing Sei1-deficient cells and mice.

## Results

### Sei1-Null Mice Are Viable and Have a Normal Longevity

**Figure 1:**
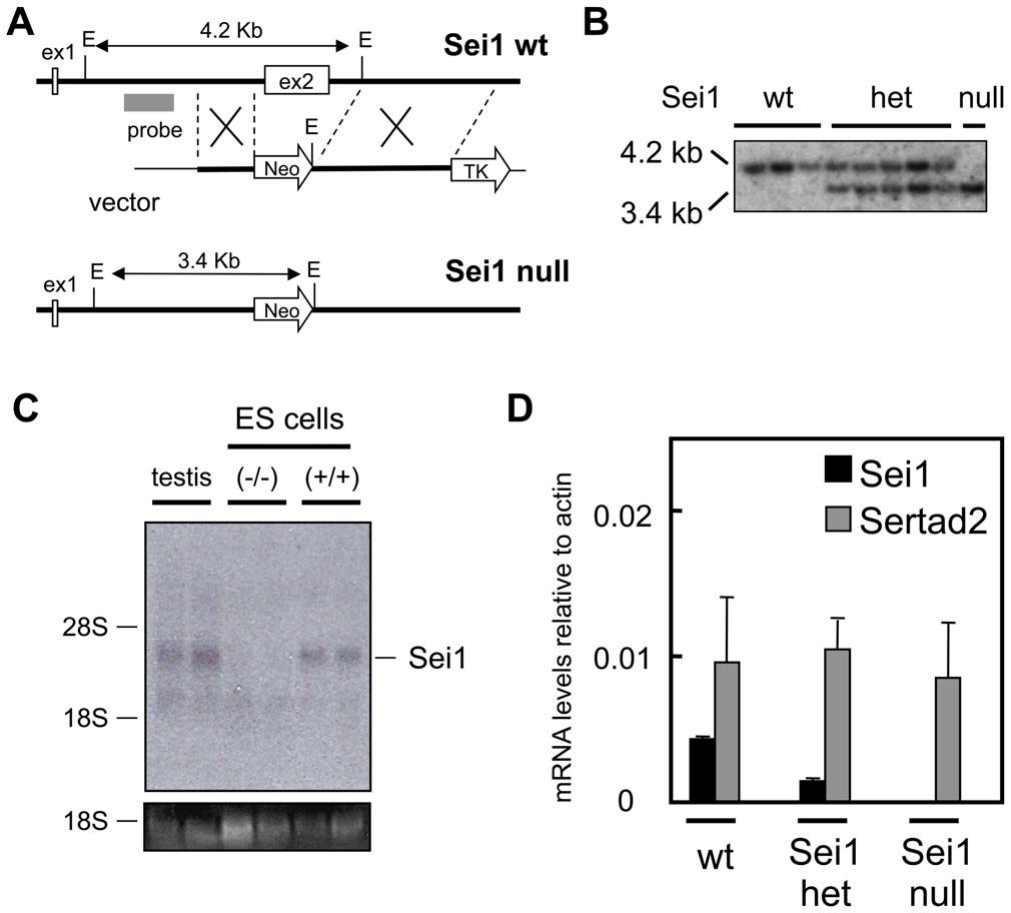
Generation of Sei1-deficient mice. *(A)* Strategy used to eliminate the second exon of Sei1, which contains the entire open reading frame for Sei1. *(B)* Southern blot confirming the ablation of Sei1 alleles in ES cells. *(C)* Northern blot confirming the loss of Sei1 transcription in Sei1-null ES. *(D)* Levels of Sei1 and Sertad2 transcripts in primary MEFs (n = 3, per genotype). Values correspond to average and s.d. of the levels relative to actin.

The Sei1 gene is composed by two exons, the first one is non-coding and the second one contains the complete open reading frame. We engineered a gene-targeting vector that replaced exon 2 of Sei1 with a neomycin resistance cassette (Fig. 1A). Targeted ES clones were confirmed by Southern blot analysis (Fig. 1B) and were used to obtain Sei1-deficient mice (see below). In addition, we generated Sei1-null ES cells by culturing Sei1-heterozygous ES cells in medium with high neomycin concentrations (see Materials and Methods; Fig. 1B). Complete Sei1 deficiency was corroborated by Northern blot analysis of Sei1-null ES cells (Fig. 1C) and qRT-PCR from Sei1-deficient MEFs (Fig. 1D). Sertad2 is the closest homolog of Sei1, and we wondered whether inactivation of Sei1 had an impact on the expression of Sertad2 in MEFs. Analysis of Sertad2 expression indicated that its levels remained unchanged in Sei1-het and Sei1-null MEFs (Fig. 1D).

Homozygous Sei1-null mice were viable, fertile and showed no overt pathologies. We checked the expression of Sei1 and Sertad2 in a variety of organs by qRT-PCR. Both Sei1 and Sertad2 were expressed in most organs examined (Supp. Fig. S1). Detailed histopathological analyses did not reveal abnormalities in Sei1-null tissues (data not shown). When wt, Sei1-het and Sei1-null cohorts of mice were aged, their survival curves were indistinguishable (Supp. Fig. S2), as well as the pathologies observed in moribund old mice (see below the incidence of spontaneous cancer).

### Sei1-Null Cells Proliferate Normally

**Figure 2 pone-0008744-g002:**
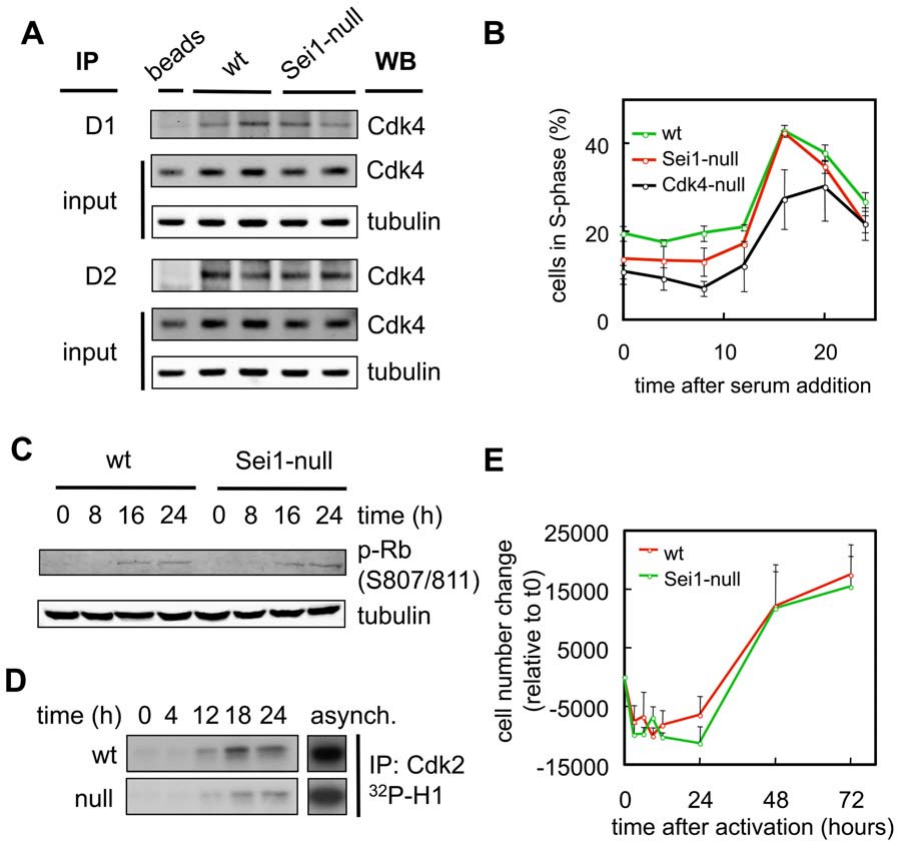
Cell cycle of Sei1-null cells. *(A)* Cell extracts from asynchronously growing MEFs of the indicated genotypes were immunoprecipitated using cyclinD1 or cyclinD2 antibodies, and then immunoblotted against Cdk4 to assess Cdk4-cyclinD complex formation. *(B, C and D)* MEF cultures of the indicated genotypes were synchronized by serum withdrawal, and forced to re-enter the cell cycle by addition of serum to the medium. *(B)* Percentage of cells in S phase was determined by the incorporation of propidium iodide and FACS analysis. The graph represents the average and s.d. of at least two independent preparations of primary MEFs per genotype. ANOVA analysis (using Sidak multiple testing) indicated that Cdk4-null MEFs were significantly different (p<0.05) than wt MEFs at all time points. Sei1-null MEFs only showed a significant difference with wt MEFs at time 8 h. *(C)* Representative example of two experiments measuring Rb phosphorylation by immunoblotting protein extracts obtained at the indicated times. *(D)* Representative example of at least two experiments assaying Cdk2 kinase activity from protein extracts of synchronyzed MEFs at the indicated times; asynch.: MEFs asynchronously growing. *(E)* Splenocytes isolated from at least three mice per genotype were activated by LPS and concanavalin A, and the number of lymphocytes was counted at the indicated times. Values correspond to the average and s.d.

Mouse embryo fibroblasts (MEFs) were obtained from wild type, Sei1-het and Sei1-null mice. First, we studied Cdk4 complexes in asynchronously growing cells, but we did not observe differences in the abundance of Cdk4/D1 or Cdk4/D2 complexes (Fig. 2A and Supp. Fig. S3A). To check whether the absence of Sei1 could affect the cell cycle, we rendered wt and Sei1-null MEFs quiescent by serum withdrawal, and triggered cell cycle re-entry by serum addition. As shown in Fig. 2B, Sei1-null MEFs entered into S phase with normal efficiency and kinetics. As a control, Cdk4-null MEFs presented a significant reduction in the percentage of cells entering S phase, as previously reported [19]. To examine in further detail the cell cycle, we checked the phosphorylation of Rb during the first cell cycle after serum re-addition, and observed no significant differences between wt and Sei1-null cells (Fig. 2C and Supp. Fig. S3B). Finally, we also assessed the activation of Cdk2 and, again, the kinetics and intensity of activation of this kinase were similar across various assays when comparing wt and Sei1-null MEFs (Fig. 2D and Supp. Fig. S3C). To widen the scope of the previous analyses, we checked cell cycle entry in splenocytes, which include both T- and B-lymphocytes. These cells remain quiescent in culture until activated with antigens. Sei1-null splenocytes entered proliferation with kinetics and efficiencies indistinguishable from wt splenocytes (Fig. 2E).

**Figure 3 pone-0008744-g003:**
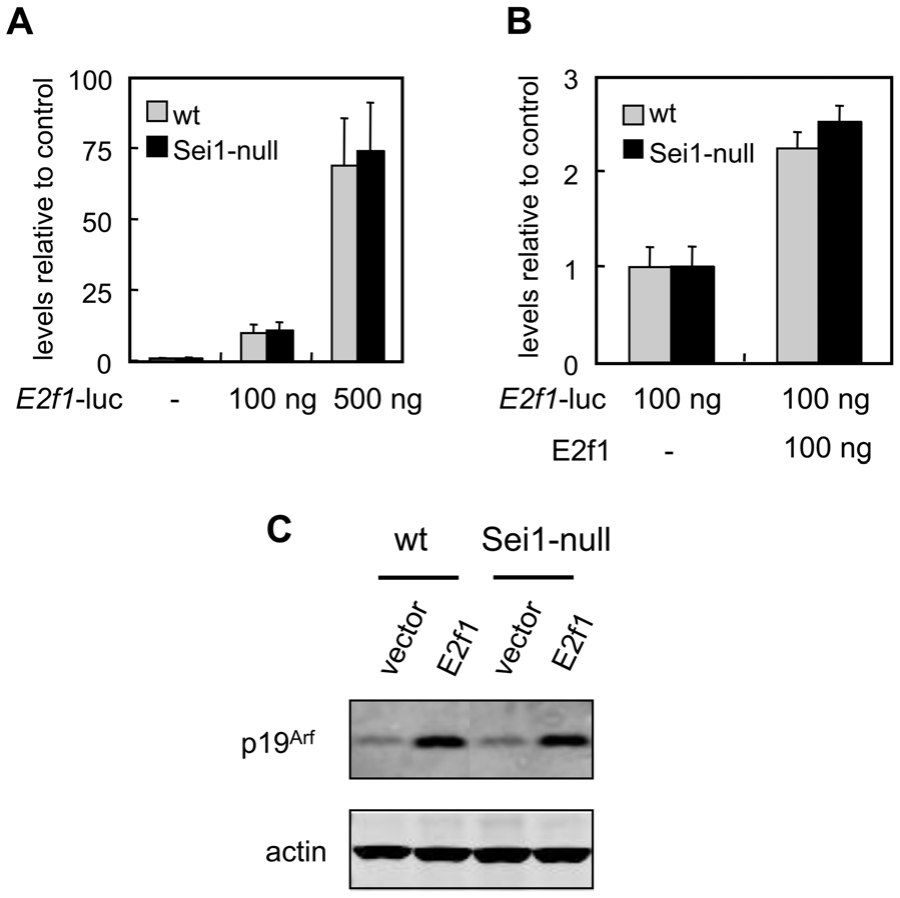
E2f1 activity in Sei1-null cells. *(A)* Endogenous E2f1 activity was measured by transfecting at least three independent preparations of primary MEFs per genotype with different amounts of a plasmid carrying the luciferase reporter under the regulation of the E2f1-responsive *E2f1* promoter. Luciferase activity was measured two days after the transfection. Values represent the average and s.d. *(B)* Ectopic E2f1 activity was measured as before after co-transfection with different amounts of a plasmid expressing E2f1. *(C)* Levels of protein p19^Arf^, a bona-fide E2f1 target, were assessed by immunoblotting of extracts from MEFs of the indicated genotypes transduced with E2f1. These results were confirmed in another independent assay. Actin was used as a loading control.

Sei1 has been also described as a positive co-factor for the transcriptional regulator E2f1 [15]. We examined *E2f1*-driven transcription using a luciferase reporter assay (based on the *E2f1* promoter, [20]). However, the absence of Sei1 did not affect the expression of luciferase by endogenous E2f1 levels (Fig. 3A) or by overexpressed transfected E2f1 (Fig. 3B). Finally, we checked the activity of E2f1 by measuring the expression of a well known E2f1 target, namely p19^Arf^ [21]. Retroviral expression of E2f1 efficiently upregulated p19^Arf^, but no differences were observed between wt and Sei1-null cells (Fig. 3C). Also, under basal conditions the levels of p19^Arf^ were similar regardless of the Sei1 genotype (Fig. 3C).

All together, we conclude that the absence of Sei1 does not affect significantly the cell cycle, neither in MEFs nor in lymphocytes, and does not impair detectably the assembly of Cdk4 complexes or the transcriptional activity of E2f1 in fibroblasts.

### Normal Immortalization and Oncogenic Transformation of Sei1-Null Cells

**Figure 4 pone-0008744-g004:**
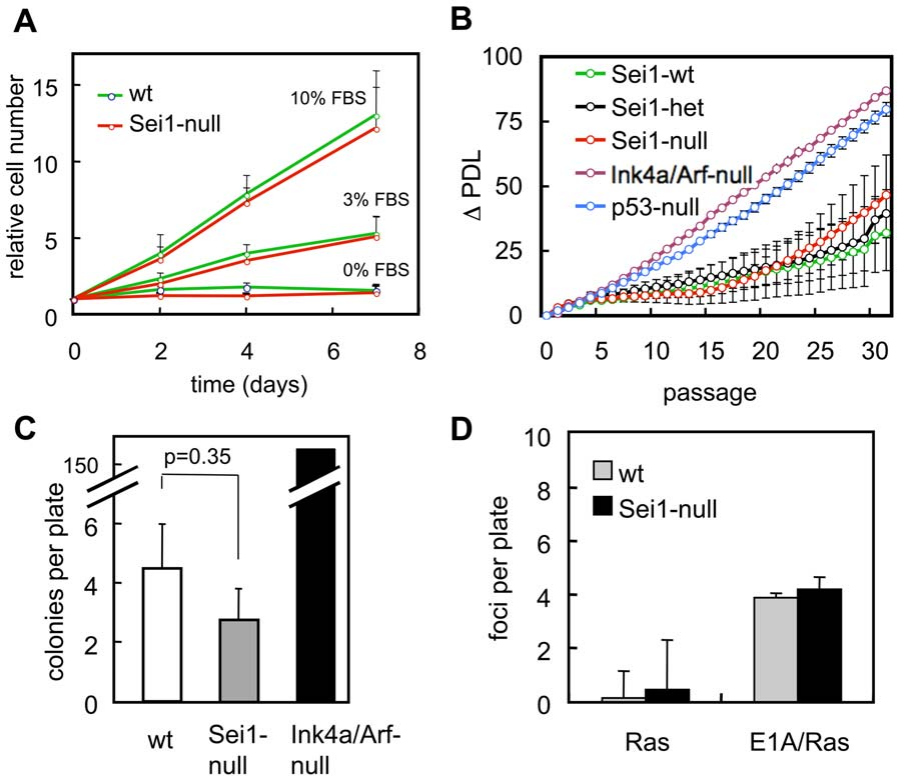
Proliferation, immortalization and transformation of Sei1-null MEFs. *(A)* Growth curves of primary MEFs grown at the indicated serum concentrations. *(B)* Senescence and immortalization were evaluated performing a 3T3 serial passage protocol of MEFs of the indicated genotype. ΔPDL, increase in population doubling level *(C)* Colony formation assay measuring the immortalization potential of MEFs of the indicated genotypes. *(D)* Foci formation assay of MEFs transduced with HRasV12 or with bicistronic E1A/HRasV12. In all the above assays, at least three independent preparations of primary MEFs per genotype were used. Values correspond to average and s.d.

Cell cycle regulators may have profound effects on the susceptibility of cells to acquire oncogenic properties [1]. Thus, we studied the serum dependence, immortalization and transformation capacity of MEFs deficient for Sei1. As shown in Fig. 4A, Sei1-null MEFs proliferate at the same rate as wt MEFs under different serum concentrations. When subjected to the 3T3 serial passage protocol, we could not observe differences in the onset of senescence or in the immortalization of wt and Sei1-null MEFs (Fig. 4B). To further test this under more stringent conditions, we seeded wt and Sei1-null cells at low cellular density and checked for the appearance of colonies. Sei1-null MEFs showed the same low colony formation capacity as wt MEFs, while Ink4a/Arf-double null MEFs, used here as a positive control, formed colonies efficiently (Fig. 4C). Finally, we examined the impact of Sei1 deficiency on the susceptibility of MEFs to neoplastic transformation by HRas^G12V^ or by the combination of HRas^G12V^ and E1A. We measured the transformation efficiency by plating infected cells at confluent density and counting the emergence of foci after two weeks in culture. We could not observe differences between Sei1-null and wt MEFs when subjected to these oncogenic transformation assays (Fig. 4D). From these data we conclude that the absence of Sei1 has no significant impact on the requirement for external growth factors, ability to immortalize spontaneously, or susceptibility to be oncogenically transformed.

### Normal Apoptosis of Sei1-Deficient Cells

**Figure 5 pone-0008744-g005:**
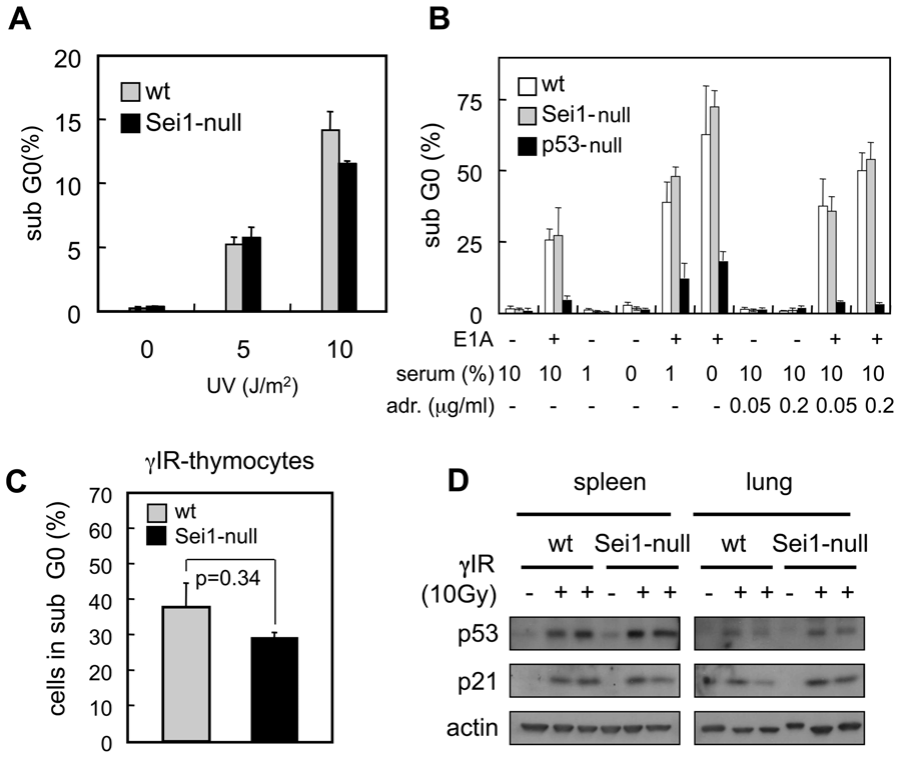
Apoptosis in Sei1-null cells. *(A and B)* Three independent preparations of primary MEFs per genotype were either irradiated with the indicated doses of UVC *(A)*, or transduced with E1A and treated with low serum conditions or with adriamycin, as indicated *(B)*. Apoptosis was measured by quantifying subG0 DNA content by FACS. *(C)* Thymocytes from three mice per genotype treated with 10 Gy of γ-radiation were isolated and apoptosis was measured 3 h post-radiation, as in (A) and (B). *(D)* Protein extracts of lung and spleen from the same irradiated mice as in (C) were immunoblotted against p53 or its target p21^Cip1^. Actin was used as a loading control.

Based on the above negative data, we decided to expand our studies to stress responses and apoptosis. First, we subjected wt and Sei1-null MEFs to different doses of ultraviolet radiation, which triggered the same levels of apoptosis in both genotypes (Fig. 5A). Then, we infected Sei1-null MEFs with the oncoprotein E1A. This oncoprotein sequesters and inhibits Rb [22] and induces a p19^Arf^/p53-dependent apoptotic response [23]. As shown in Fig. 5B, expression of E1A induced apoptosis in wt and Sei1-null MEFs to similar levels, while p53-null MEFs were not affected. E1A sensitizes MEFs to other apoptotic stimuli, such as serum withdrawal or adriamycin [23]. However, Sei1-null MEFs infected with E1A were as sensitive to serum withdrawal and adriamycin as were wt MEFs (Fig. 5B). To extend our study to other cell types, we irradiated mice with ionizing radiation and measured apoptosis in thymocytes, however, there were no significant differences between Sei1-null and wt thymocytes (Fig. 5C). Finally, we analyzed the response of p53 and its target p21^Cip1^ to ionizing radiation in the spleen and the lung. As observed in Fig. 5D, both proteins, p53 and p21, were clearly induced upon irradiation but this induction was not affected by the status of Sei1. Also, basal levels of p21^Cip1^ and p53 were not detectably higher in Sei1-null tissues compared to wt ones. We conclude that Sei1 does not have a significant impact on the response of p53 to DNA damage and the susceptibility to undergo apoptosis.

### Sei1-Deficiency Does Not Impact on Tumor Development in Mice

**Figure 6:**
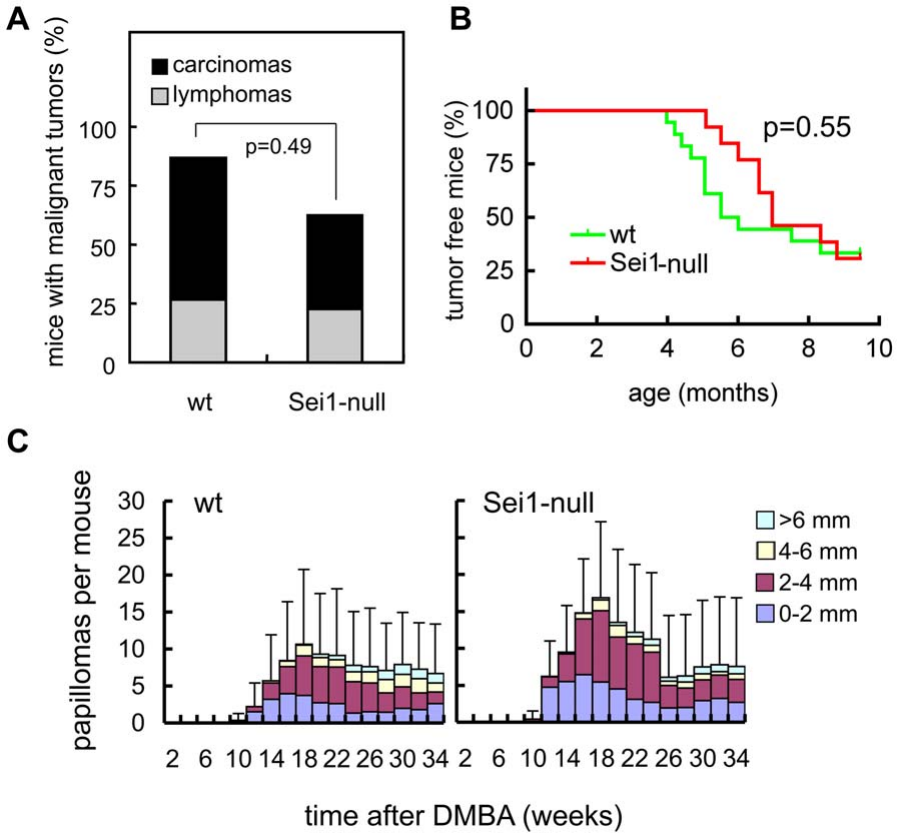
Cancer susceptibility in Sei1-null mice. *(A)* Spontaneous cancer incidence and spectrum in old moribund mice (n = 15 for wt mice, and n = 26 for Sei1-null mice). Presence and type of cancer was determined upon detailed histopathological analyses by one of the authors (J.M.F.). Fisher's exact test was used to determine statistical differences in total cancer incidence. *(B)* Latency of fibrosarcoma development after intramuscular injection with 3MC represented by Kaplan-Meier curves (n = 16 for wt mice, and n = 13 for Sei1-null mice). Statistical analysis was performed using the Logrank test. *(C)* Papilloma latency, incidence and size after DMBA/TPA tumorigenesis in mice of the indicated genotypes (n = 14 per genotype). Bars correspond to the s.d. of the average number of total papillomas per mouse. Student's t-test was used to compare the two genotypes but no significant differences were found.

To address the impact of Sei1 on tumorigenesis, we first focused on the emergence of spontaneous malignant tumors with aging. Sei1-null mice developed spontaneous malignant tumors with the same incidence and spectrum as wt mice (Fig. 6A). To further explore this issue, we decided to subject our mice to carcinogenic treatments. For this, we performed a carcinogenic assay that consisted in intramuscular injection of 3-methyl-cholanthrene (3MC), which results in the development of fibrosarcomas. We could not observe significant differences between the onset of fibrosarcomas in wt and Sei1-null mice (Fig. 6B). We then performed a classical skin carcinogenesis protocol based on initiation by DMBA and promotion by TPA. Again, Sei1-null mice developed papillomas with similar kinetics and incidence as wt mice (Fig. 6C). No significant differences could be observed between the number of papillomas or their size; also no conversion to carcinoma was observed in any genotype during the 34 weeks of observation. In summary, Sei1-ablation does not seem to have a major effect on the development of spontaneous or chemically-induced tumors in mice.

### Defective Pancreatic β-Cell Function in Sei1-Deficient Mice

**Figure 7:**
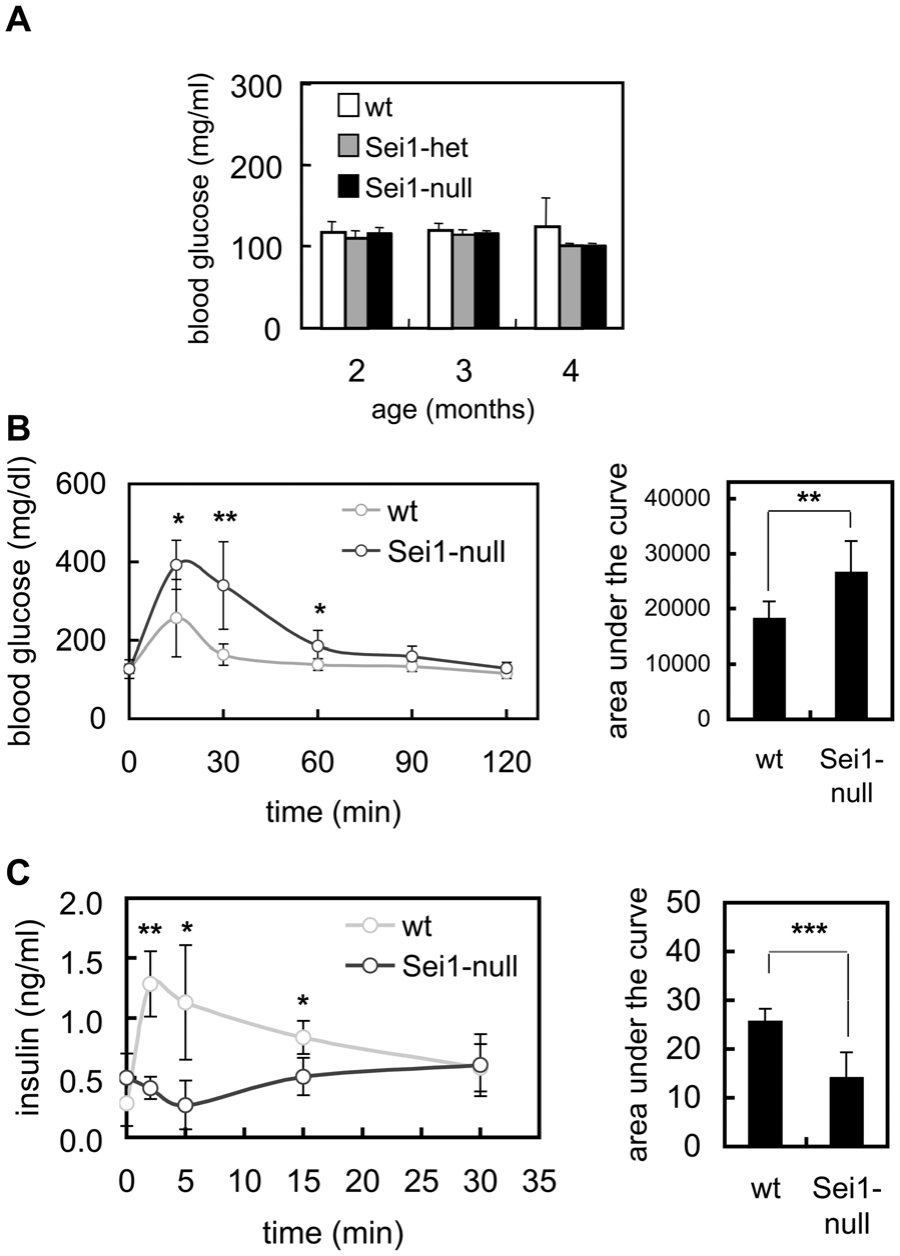
Glucose homeostasis in Sei1-null mice. *(A)* Blood glucose levels were measured in groups of at least 4 male mice with the indicated genotypes maintained under *ad libitum* feeding. *(B)* Glucose tolerance test (GTT) on male mice (n = 8 per genotype), of 8 weeks of age. The area under the curve (AUC) for each genotype is represented to the right. *(C)* Insulin release test (IR) and its AUC performed in the same mice as in B (one week after the GTT). Values correspond to the average and s.d. Statistical differences were determined by the Student's t-test; *p<0.05; **p<0.01; and ***p<0.001.

Genetic ablation of Cdk4 activity, cyclin D2, or combined E2f1 and E2f2, results in defective maintenance of adult pancreatic β-cells, eventually resulting in severe diabetes [5]–[7]. Considering the implication of Sei1 in the positive regulation of the above-mentioned set of proteins (see Introduction), we wondered whether Sei1-null mice could have mild, but detectable, defects in pancreatic islets. For this, we first measured blood glucose levels of wt, Sei1-het and Sei1-null mice during the first months of life, but no significant differences were observed in basal glycemia under conditions of *ad libitum* feeding (Fig. 7A). Interestingly, when subjected to the glucose tolerance test, Sei1-deficient mice showed a poorer clearance of glucose from blood compared to wt mice, both under standard diet (Fig. 7B) or under high fat diet (Supp. Fig. S4A). When insulin release was measured after injection of glucose, Sei1-null mice showed a dramatically impaired secretion of insulin to the bloodstream (Fig. 7C). Finally, sensitivity to insulin was not altered in the absence of Sei1 (Supp. Fig. S4B). Together, the above data point towards a specific defect in pancreatic β-cell function in Sei1-null mice.

**Figure 8:**
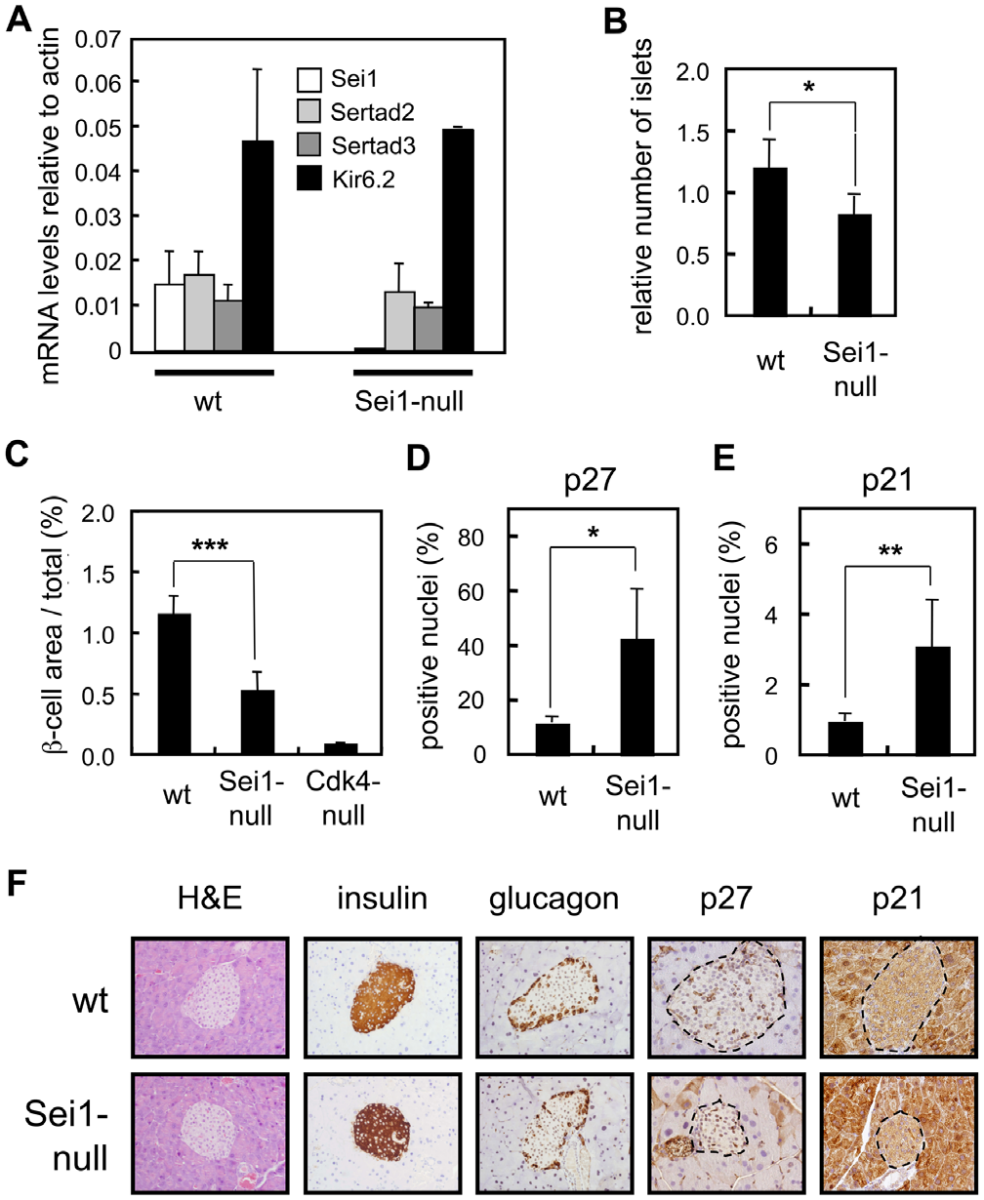
Pancreatic islets in Sei1-null mice. (*A*) Levels of mRNA expression of the Sertad family members Sei1, Sertad2 and Sertad3, as well as, the pancreatic islet gene Kir6.2, were measured using qRT-PCR in total mRNA from isolated pancreatic islets of wt (n = 4) and Sei1-null (n = 2) male mice. *(B–F)* Paraffin sections of pancreas (n = 4 per genotype) of the indicated genotypes were stained with antibodies against insulin (*B* and *C*), p27^Kip1^ (*D*) or p21^Cip1^ (*E*). *(B)* Number of islets relative to the total area of the pancreas. *(C)* Ratio between the β-cell islet area and the total pancreas area. *(D)* p27^Kip1^- or *(E)* p21^Cip1^-positive nuclei relative to total β-cell area. *(F)* Representative examples of the indicated stainings. The dotted lines mark the limits of the pancreatic islets. Values correspond to the average and s.d. Statistical differences were determined by the Student's t-test; *p<0.05; **p<0.01; and ***p<0.001.

To further reinforce a role of Sei1 in β-cells, we evaluated the expression of Sei1, Sertad2 and Sertad3 in isolated pancreatic islets (being Sertad2 the closest paralog of Sei1). Interestingly, the mRNA levels of Sei1, Sertad2 and Sertad3 were similar in wt islets, and no compensatory changes in Sertad2 or Sertad3 were observed in Sei1-null islets (Fig. 8A). As a control for the isolation of islets, we detected the transcript for Kir6.2 (also known as Kcnj11), which is expressed in the islets but not in the exocrine pancreas [24] and no changes were observed associated to the status of Sei1 (Fig. 8A). Of note, compared with a large set of tissues (a total of 17 tissues), islets presented the highest levels of Sei1 expression (Suppl. Fig. S1). Moreover, the ratio between the levels of Sei1 and Sertad2 mRNAs was also maximal in the islets (Suppl. Fig. S1). Therefore, it can be concluded that the levels of Sei1 in islets are uniquely high.

In recent years, it has been established that Cdk4/cyclinD2 plays an important role in the maintenance of β-cells noticeable after 3 months of age [5]–[7]. We wondered whether Sei1-null mice could have a similar defect. We measured the size of the pancreatic islets in 4- to 6-months old mice of wt, Sei1-null and Cdk4-null genotypes. Interestingly, pancreatic islets of Sei1-null mice showed a significant reduction in the number of pancreatic islets (Fig. 8B), as well as, a decrease of approximately 50% in β-cell area (Fig. 8C). The reduction in β-cell surface observed in Sei1-null mice was of intermediate magnitude when compared with the severe defect characteristic of Cdk4-null mice (Fig. 8C). Sei1-null islets presented a normal architecture, with α-cells predominantly located at the periphery of the islet and β-cells in the core (see below Fig. 8F). In an effort to understand the mechanisms that lead to decreased β-cell surface in the absence of Sei1, we measured apoptosis and proliferation by TUNEL and Ki67 immunohistochemistry, respectively, however the number of positive islet cells for both markers was very low (<1%) and quantifications could not detect significant differences between wt and Sei1-null islets (data not shown). In an alternative approach, immunohistochemical detection of the cell cycle inhibitors p21^Cip1^ and p27^Kip1^ revealed higher levels of positive nuclei in Sei1-null pancreatic islets compared to wt islets (Fig. 8D–F). Interestingly, the mRNA levels of p21^Cip1^ and p27^Kip1^ were similar in the islets, as were the protein levels of p53 and p27^Kip1^ (Supp. Fig. S5). These observations suggest that the increase in nuclei positive for p21^Cip1^ and p27^Kip1^ is due to an altered balance in the subcellular localization of these proteins. From these data, we conclude that Sei1 plays a relevant role in the maintenance and function of pancreatic β-cells. Absence of Sei1 results in higher levels of the cell cycle inhibitors p21^Cip1^ and p27^Kip1^, reduced β-cell area, decreased insulin secretion and, consequently, glucose intolerance.

## Discussion

Sei1 has been involved in the positive regulation of the cell cycle and proliferation [10], [15]–[17] and, accordingly, its expression is upregulated in several types of tumors [16], [18], [25]. Experimental overexpression of Sei1 can provoke hyperproliferation [10], genomic instability [17] and inhibition of apoptosis [26]. In an effort to understand the role of Sei1 in the context of the organism, here we have generated and characterized Sei1 deficient mice.

Experiments with primary fibroblasts and lymphocytes derived from Sei1-null mice did not reveal any cellular or molecular alteration regarding proliferation, immortalization, oncogenic transformation, and apoptotic responses to DNA damage. Furthermore, in the case of primary fibroblasts, Cdk4 complexes, Cdk2 activity, Rb phosphorylation, E2f transactivation and p53 responses were normal in the absence of Sei1. At the organismal level, Sei1-null mice were viable and showed no overt phenotypes, including normal lifespan, spontaneous tumors and chemically-induced tumors. The most probable explanation for this lack of phenotypes is redundancy with other members of the Sertad family that compensate for the absence of Sei1. Sertad2 is the closest relative to Sei1 and, indeed, Sei1 and Sertad2 share functional properties, including their ability to stimulate E2f1 activity [15]. Sei1 is also able to interact with Cdk4 and stimulate its activity [10], although at present it remains unknown whether this activity is shared or not with the other members of the Sertad family.

A common feature of a number of mouse models deficient for proteins in the Cdk4/cyclinD/E2f pathway is a defective glucose homeostasis due to an impaired maintenance of pancreatic β-cells [5]–[8]. The expression levels of Sei1, but not of Sertad2, are maximal in pancreatic islets compared to any other tissue examined (Supp. Fig. S1); and, moreover, Sertad2 and Sertad3 mRNA levels are not altered in Sei1-null pancreatic islets. All these observations suggest that pancreatic islets could be more particularly sensitive to the absence of Sei1. Interestingly, Sei1-null mice displayed abnormal insulin secretion and blood glucose clearance, as well as a reduction in pancreatic β-cell surface. In addition, pancreatic islets of Sei1-null mice showed increased nuclear staining of the cell cycle inhibitors p27^Kip1^ and p21^Cip1^. These two cell cycle inhibitors have been implicated in the negative control of β-cell mass [8]. In particular, overexpression of p27^Kip1^ in islets results in decreased islet mass and diabetes [27]. Similarly, mice deficient in Skp2, which is responsible for the normal degradation of p27^Kip1^, present ubiquitous higher levels of p27^Kip1^, decreased β-cell mass and glucose intolerance, and all these phenotypes are rescued in Skp2/p27^Kip1^-doubly deficient mice [28]. At present, we do not know why p27^Kip1^ and p21^Cip1^ accumulate in the nuclei of Sei1-null β-cells. However, it is tempting to speculate that the absence of Sei1 may increase the availability of Cdk4/D complexes that can interact with p27^Kip1^ or p21^Cip1^, thus increasing the amount of Cdk4/D/(p27^Kip1^-p21^Cip1^) complexes, which normally reside in the nucleus [12], [13]. Although still under debate [29], recent evidence indicates that Cdk4/D/(p27^Kip1^-p21^Cip1^) complexes are inactive [14] and this would be consistent with the decreased islet size of Sei1-null mice.

We conclude that Sei1 ablation in mice does not affect tumor growth, but the germline-based strategy followed in this work does not preclude possible effects of acute Sei1 ablation in cancer maintenance. As for the glucose homeostasis, our results suggest that Sei1 may play an important role in diabetes or metabolic disorders.

## Materials and Methods

### Ethics Statement

Mice were treated in accordance with the Spanish Laws and the Guidelines for Humane Endpoints for Animals Used in Biomedical Research, The Spanish National Cancer Research Center (CNIO) is part of the “Carlos III” Health Institute (ISCIII) and all protocols were previously subjected and approved by the Ethical Committee of the ISCIII.

### Generation of Sei1-Deficient Mice and Animal Care

Mice genetically deficient for Sei1 were produced using standard procedures. The construct that eliminates exon 2 of Sei1 (Fig. 1A) was linearized and electroporated into R1 embryonic stem (ES) cells (129Sv). One ES cell clone was used to produce chimaeric male mice, which were crossed with Balb/c females, and the progeny from this cross was genotyped by Southern blotting. All the assays reported in Figs. 1–6 were performed with mice of mixed genetic background Balb/c;129Sv (50∶50). All the assays reported in Figs. 7 and 8 were performed in mice that had been backcrossed 9 times with C57BL/6 and therefore had a >99.9% C57BL/6 genetic background. Animals were housed at the CNIO, inside their corresponding pathogen-free barrier areas. All mice were monitored twice per week, and moribund mice were killed humanely. Tumors from non-autolysed tissues were recovered from moribund or recently deceased mice. Tissue samples were fixed in 10% buffered formalin, embedded in paraffin wax, sectioned at 4 mm and stained with haematoxylin and eosin (H&E) for a detailed histopathological analysis.

### Antibodies

The antibodies used for immunoblotting and immunoprecipitation were: Cdk4 (C-22, sc-260), Cdk2 (M-2, sc-163), cyclin D1 (C-20, sc-717), cyclin D2 (M-20, sc-593), p21^Cip1^ (C-19-G, sc-397-G), and p19^Arf^ (5-C3-1, sc-32748), all from Santa Cruz; and p53 (CM-5), from Novocastra. The antibodies used for immunohistochemistry were: p21^Cip1^ (C-19-G, sc-397-G), from Santa Cruz; p27^Kip1^ (DCS-72.F6), from Thermo Scientific; insulin (#A564), from Dako; and glucagon (K79bB10), from Sigma Aldrich.

### Cell Culture

MEFs were isolated from embryos at 13.5 days post-coitum from crosses between Sei1(+/−) mice of mixed genetic background Balb/c;129Sv (50∶50), and were cultured in Dulbecco's modified Eagle's medium (DMEM; GIBCO) supplemented with 10% Fetal Bovine Serum (FBS, Hyclone) and antibiotic/antimycotic (GIBCO).

To obtain splenocytes and thymocytes, spleens were disaggregated using a cell strainer (Nunc), and resuspended in complete medium, comprising RPMI supplemented with 100 mM Hepes buffer pH 7.5 (BioWhitaker), 2 mM L-Glutamine (GIBCO), 1 mM sodium pyruvate (GIBCO), a commercial mix of non-essential amino acids (BioWhitaker), 0.1% β-mercaptoethanol (Sigma) and antibiotic/antimycotic (GIBCO). Red blood cells were lysed by incubating the preparation for 10 minutes at 4°C in 10 mM KHCO_3_, 150 mM NH_4_Cl, 0.1 mM EDTA pH 8, all taken to pH 7.3. Thymocytes were frozen or processed for FACS analysis at this point, whereas splenocytes were plated in complete medium at a density of 2×10^6^ cells/ml, activated with 20 µg/ml LPS and 5 µg/ml concanavalin A (Sigma), and counted at different time points.

For cell cycle analysis, 2×10^6^ were plated in 10 cm diameter dishes and left to reach confluency for 3 days; then, cells were washed 3 times with PBS and cultured in medium without serum for another 3 days. After this time medium with 10% FBS was added, and cells were processed for FACS analysis or protein extraction at different times.

For luciferase assays, cells were transfected with pGL3-Basic (Promega) without promoter (basal transcription control) or with the human E2f1 promoter cloned upstream the luciferase reporter. pBabe-E1Fα-Renilla was used as an internal transfection control. Two days later cells were processed using the Dual Luciferase Assay System (Promega) following the manufacturer's instructions, and firefly and Renilla luciferase activities were measured in a luminometer Glomax (Promega). Firefly luciferase values were normalized to the Renilla control values.

For growth curves, 1.5×10^5^ cells were plated in 35 mm diameter multiwell plates, cultured in media with different serum concentrations and counted 4 hours later (time point 0), or 2, 4 or 7 days after plating.

Serial 3T3 cultivation was done as described [30]. Briefly, 10^6^ cells were plated on 10 cm diameter dishes, 3 days later the total number of cells in the dish were counted, and 10^6^ cells were replated again. This procedure was repeated for 40 passages. The increase in population doubling level (ΔPDL), was calculated according to the formula ΔPDL = log(n_f_/n_0_)/log2, where n_0_ is the initial number of cells and n_f_ is the final number of cells.

Colony and foci formation assays were performed as described elsewhere [31]. For colonies formation, 6×10^3^ cells were distributed on a total of three dishes of 10 cm diameter and incubated in DMEM plus 10% FBS. After 2 weeks of incubation with regular changes of medium every 3 days, dishes were stained with Giemsa, and the number of visible colonies (>1.5 mm of diameter) was scored.

For foci formation, early-passage MEFs (P0-P1) derived from individual embryos were seeded (10^6^ cells) in plates of 10 cm diameter and cultured overnight in complete medium containing 10% FBS. The medium was changed 1 h before transfections began. For these assays, we used oncogenic H-rasV12 under an LTR promoter in vector pBabe-puro. Transfections were done by a standard calcium-phosphate procedure with 10 mg of pBabe-puro-H-rasV12 plus 20 mg of carrier DNA plasmid (empty pBabe-puro). After 8 h of incubation with the precipitates, the incubation medium was changed and, 18 h later, cells were distributed equally into three 10 cm diameter plates. Dishes were refed with fresh medium every 3 days. At day 21 post-transfection, cells were fixed and stained with Giemsa, and the total number of foci in the three plates was scored.

For retroviral transduction, plasmids indicated in each experiment were co-transfected along with pCL-Eco (to produce the ecotropic variant of the Moloney murine leukemia virus) into 293T cells using Fugene 6 (Roche) as reagent, following the manufacturer's instructions. After two days the supernatant containing the viral particles was filtered by 45 µm pore-filters, mixed with polybrene to a final concentration of 4µg/ml and added to 10 cm diameter plates with the recipient MEFs. This process was repeated three more times, after which infected MEFs were cultured for 2 days in medium with 2 µg/ml puromycin for selection of the infected cells.

For induction of apoptosis, cells were either washed twice with PBS and irradiated with a UVC irradiator (Hoeffer) or transduced with viral E1A and subjected to low serum concentration or 0.2 or 0.05 µg/ml adriamycin for two days, after which they were processed for FACS analysis.

### FACS Analysis

Cells were trypsinized and resuspended in cold PBS. Cold ethanol was added dropwise under constant agitation to this cell suspension until reaching a 70% final concentration, and the preparation was kept at 4°C for one to 7 days. These cells were resuspended in PBS containing 30 µg/ml of propidium iodide (Sigma) and 0.1 mg/ml RNase (Invitrogen), incubated at 37°C for 30 minutes, and analyzed by flow cytometry using FACScalibur (BD Biosciences). CellQuest Pro (BD Biosciences) software was used to measure subG0 apoptotic bodies, and the Modfit (Verity Software House) software to obtain cell cycle profiles.

### Immunoprecipitations and Kinase Assays

Protein lysates obtained with the NP-40 buffer (0.5% NP-40, 1% sodium deoxycholate, 0.1% SDS, 150 mM NaCl, 10 mM phosphate buffer pH 7.2) were pre-cleared by incubating them with protein A or G (Santa Cruz). Supernatants were incubated O/N at 4°C with 5–10 µg antibodies against cyclin D1, cyclin D2 or Cdk2 using orbital agitation, after which protein A or G was added and the mix was incubated at 4°C for 2 hours. Supernatant was kept for the loading control, and the beads were washed three times with the NP-40 buffer. For the IPs, the beads were resuspended in 50 µl of Laemmli buffer, boiled for 5 minutes and immunoblotted using an antibody against Cdk4. For the Cdk2 kinase assays, the beads were washed three times with the kinase assay buffer (20 mM Tris-HCl pH 8; 10 mM MgCl_2_; 1 mM EGTA; 1 mM DTT; 1 mM PMSF; 1 mM Na_3_VO_4_; 1 mM NaF and a cocktail of protease inhibitors (Roche)). The beads were then incubated with a mix of 20 µl of the kinase buffer, 5 µg of histone H1 (Boeringer), 1 ml of 0.5 µM cold ATP and 10 µCi of γ^32^P-ATP at 30°C for 30 minutes, mixed with 100 µl of Laemmli loading buffer, loaded into a gel and developed by autoradiography.

### Quantitative RT-PCR

Quantitative PCRs of the indicated genes was performed using the 7900Fast qRTPCR system (Applied Biosystems), and data were analyzed using the SDS software provided with it. The oligonucleotides used were the following (listed 5′ ->3′): Sertad1: sense, CAAGCGGGAGGAGGAGGAGACGAT; antisense, AGAAGGGGCTGGGGGCTGGAT; Sertad2: sense, CGCACAGATGACTCACGATT; antisense, GGGGTCAAAATCGTACATGG; Sertad3: sense, CAGGCTTGTCGGAGGTAGTC; antisense, TTGGACTCCAGTCCCACTTC; Kir6.2: sense, AGAATATCGTCGGGCTGATG; antisense, CTCTTTCGGAGGTCCCCTAC; actin, sense, GGCACCACACCTTCTACAATG; antisense, GTGGTGGTGAAGCTGTAGCC.

### Chemical Carcinogenesis

For induction of fibrosarcomas, mice of 3–5 months received 1 mg of 3-methyl-cholanthrene (Sigma-Aldrich) in 50 µl of sesame oil by intramuscular injection in the right leg. Mice were examined twice per week until the tumor was palpable and scored in Kaplan-Meier graphs.

For induction of papillomas, mice of 2–4 months received a single dose of 20 µg DMBA dissolved in 200 µl of acetone in their shaved backs. Five days later started the treatment with 12.5 µg of TPA dissolved in 200 µl of acetone twice a week for 19 weeks. The number of papillomas was counted, and their size measured with a Vermier caliber during 34 weeks.

### Glucose Homeostasis and Analysis of Pancreatic Islets

To measure pancreatic islets, paraffin sections of pancreas were stained with an insulin antibody (DAKO), and the surface ratios of β-pancreatic islets versus total pancreas were measured using a Mirax scan, and analyzed using the AxioVision software, both from Zeiss. To measure glucose levels, blood samples from male mice fed *ad libitum* were analyzed for their glucose content using Glucocard Memory 2 Base (A. Menarini Diagnostics). For the high fat diet, mice were fed with Research Diets 12451, 45% kJ from fat, during two months. For the glucose tolerance test (GTT), insulin tolerance test (ITT) and insulin release (IR) assays, groups of wt or Sei1-null males (n = 8 per genotype) of 9 weeks of age were used and all the assays begun after overnight starvation. For the GTT, mice were injected intraperitoneally with a 50% solution of dextrose in PBS and a total amount of 2 g of dextrose per Kg of mouse weight, and blood glucose was measured at 0, 15, 30, 60, 90 and 120 minutes. For the ITT assay, mice were injected with 0.75 U/Kg insulin in saline, and blood glucose was measured at 0, 15, 30, 45, 60 and 90 minutes. For the IR assay, mice were injected with 3 g/Kg dextrose in PBS, and blood samples were obtained at 0, 2, 5, 15 and 30 minutes. Serum from these samples were analyzed for their insulin content using the Ultra Sensitive Mouse Insulin ELISA Kit (Crystal Chem Inc) and blood glucose was also monitored to control that the assay was correctly performed.

Pancreatic islets were isolated following standard methods, essentially as described in [32].

### Statistics

Data are presented as mean and standard deviation. Unless otherwise stated, statistical analyses were performed using unpaired Student's *t*-test. Differences were considered statistically significant at *p*<0.05. The following symbols are used: *p<0.05; **p<0.01 and ***p<0.001.

## Supporting Information

Figure S1 Tissue distribution of Sei1 and Sertad2 expression. (Top) Total RNA from a panel of mouse organs was isolated and analyzed by qRT-PCR for the expression of Sei1 relative to actin. (Middle) Sertad2 mRNA levels analyzed as before relative to actin. (Bottom) Ratio between Sei1 and Sertad2 expression levels.
(0.40 MB TIF)

Figure S2 Survival curves of Sei1-null mice. Cohorts of 17 males and 16 females for the wt group, and 22 males and 23 females for the Sei1-null group, were aged until signs of terminal morbidity. Data are represented as Kaplan-Meier curves. Logrank test indicated that differences were not statistically significant.
(0.57 MB TIF)

Figure S3 Cell cycle of Sei1-null cells. (A) Quantification of the experiment shown in main Fig. 2A (n = 2). (B) Quantification of the experiment shown in main Fig. 2C (n = 2). (C) Quantification of the experiment shown in main Fig. 2D (n = 2) Values correspond to the average and s.d. Student's t-test was used to compare the two genotypes but no significant differences were found.
(0.29 MB TIF)

Figure S4 Pancreatic function of Sei1-deficient mice. (A) Glucose tolerance test (GTT) performed in male mice (n = 8 per genotype) after two months feeding with a high fat diet. The area under the curve (AUC) is represented to the right. (B) Insulin tolerance test (ITT) performed in the same mice of Figure 7B and 7C of the main paper.
(0.26 MB TIF)

Figure S5 Levels of cell cycle regulators in Sei1-null pancreatic islets. Islets were isolated from wt and Sei1-null mice (n = 3 for each genotype) and each individual preparation was analyzed separately. RNA levels were measured by quantitative real-time PCR and protein levels by immunoblot (the inset shows one example per genotype). Student's t-test was used to compare the two genotypes but no significant differences were found.
(0.32 MB TIF)
