# Supplementary figures and images for: Normal Proliferation and Tumorigenesis but Impaired Pancreatic Function in Mice Lacking the Cell Cycle Regulator Sei1
Source: PLoS One. 2010 Jan 18;5(1):e8744. doi: 10.1371/journal.pone.0008744 (PMC2807453; doi:10.1371/journal.pone.0008744)

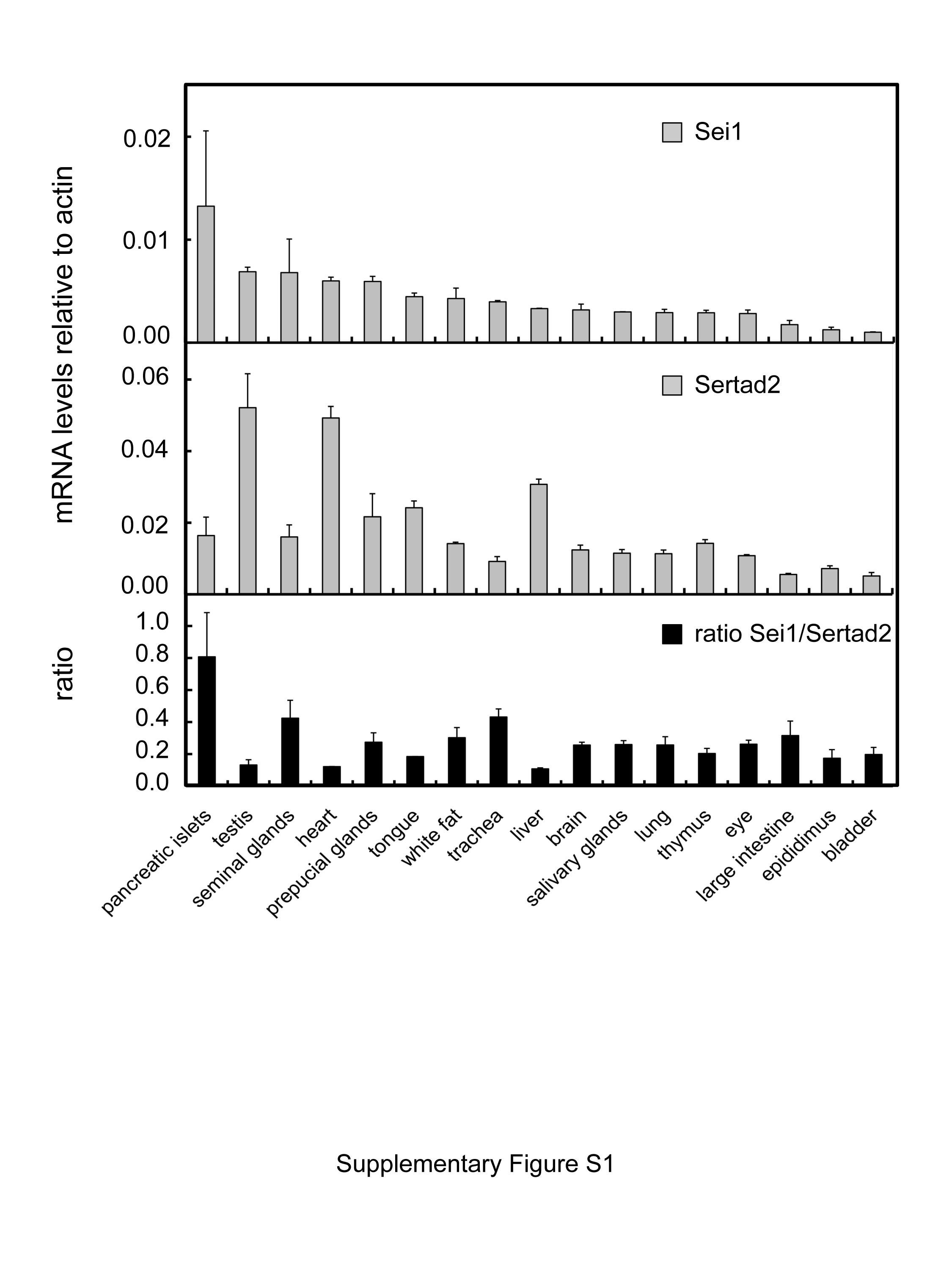

Supplement: Figure S1 — Tissue distribution of Sei1 and Sertad2 expression. (Top) Total RNA from a panel of mouse organs was isolated and analyzed by qRT-PCR for the expression of Sei1 relative to actin. (Middle) Sertad2 mRNA levels analyzed as before relative to actin. (Bottom) Ratio between Sei1 and Sertad2 expression levels. (0.40 MB TIF) [file pone.0008744.s001.tif]

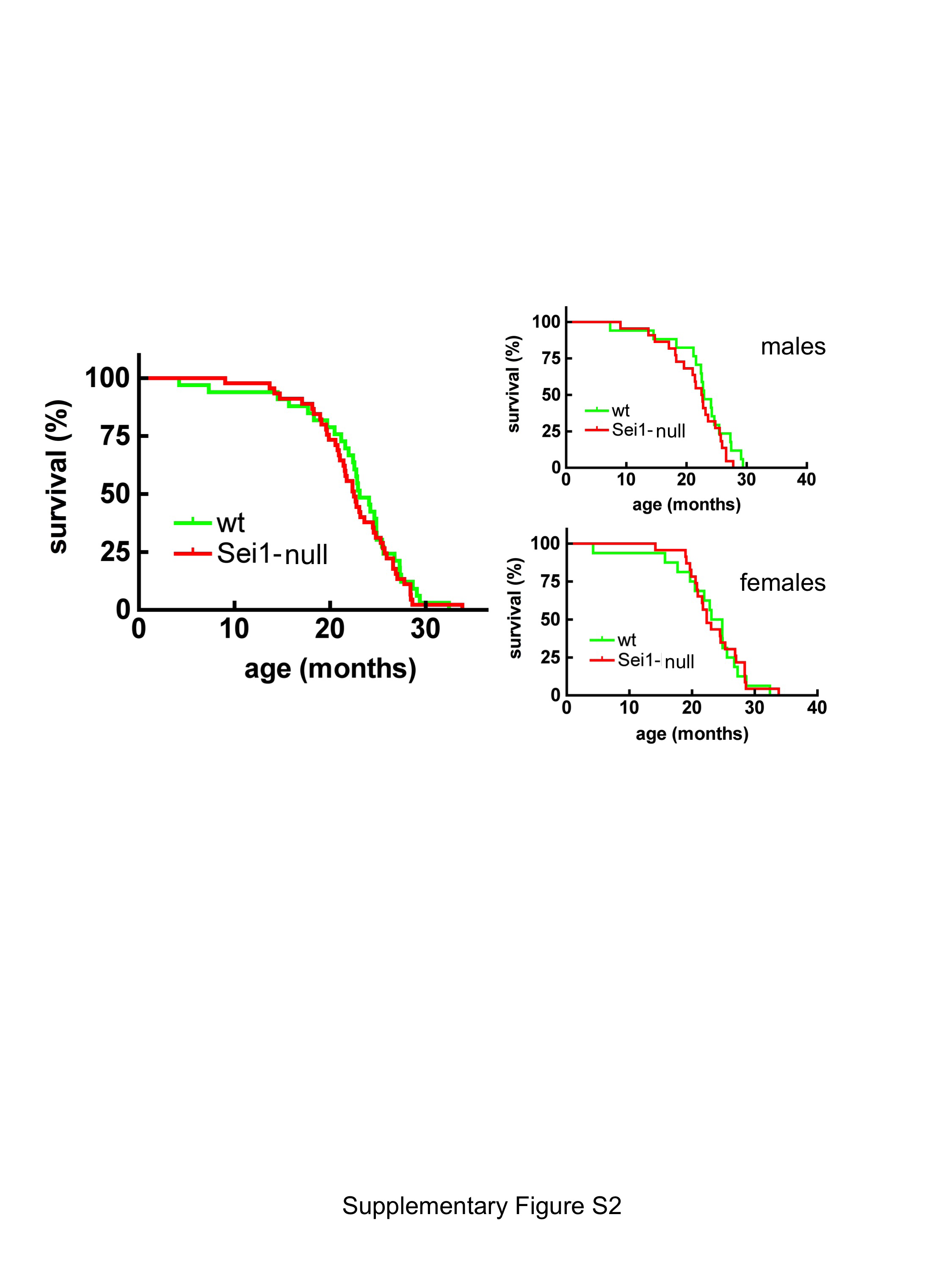

Supplement: Figure S2 — Survival curves of Sei1-null mice. Cohorts of 17 males and 16 females for the wt group, and 22 males and 23 females for the Sei1-null group, were aged until signs of terminal morbidity. Data are represented as Kaplan-Meier curves. Logrank test indicated that differences were not statistically significant. (0.57 MB TIF) [file pone.0008744.s002.tif]

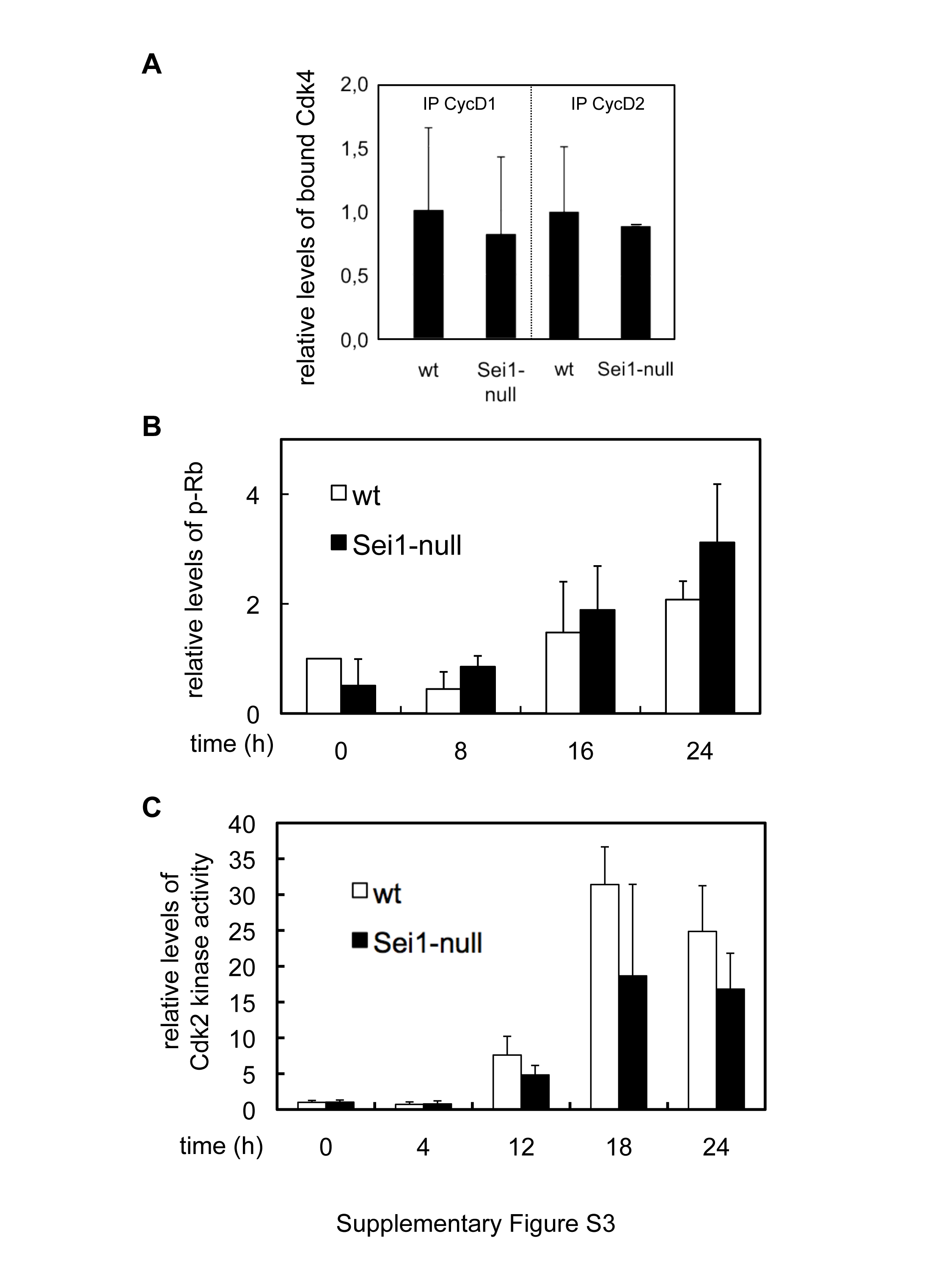

Supplement: Figure S3 — Cell cycle of Sei1-null cells. (A) Quantification of the experiment shown in main Fig. 2A (n = 2). (B) Quantification of the experiment shown in main Fig. 2C (n = 2). (C) Quantification of the experiment shown in main Fig. 2D (n = 2) Values correspond to the average and s.d. Student's t-test was used to compare the two genotypes but no significant differences were found. (0.29 MB TIF) [file pone.0008744.s003.tif]

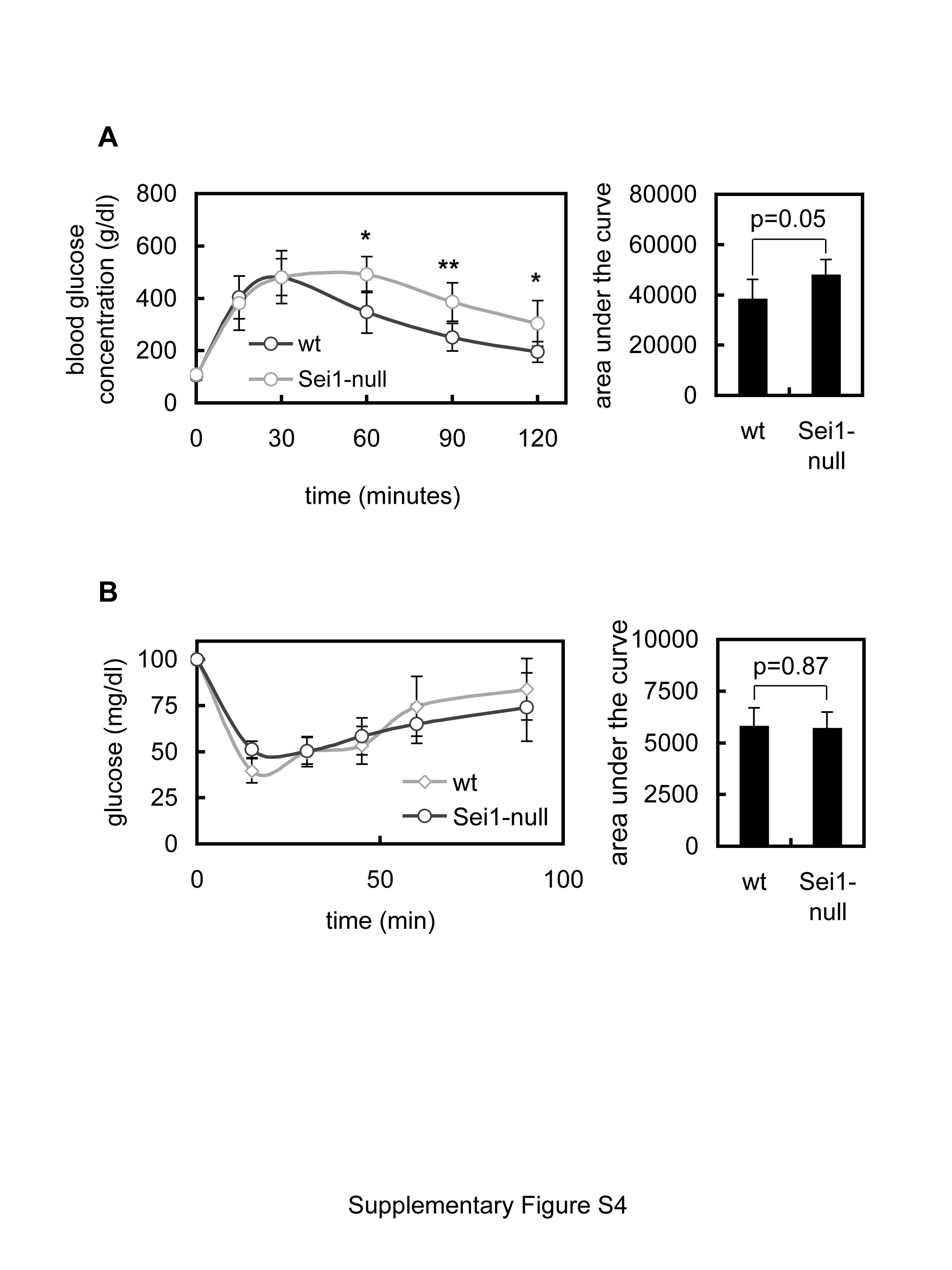

Supplement: Figure S4 — Pancreatic function of Sei1-deficient mice. (A) Glucose tolerance test (GTT) performed in male mice (n = 8 per genotype) after two months feeding with a high fat diet. The area under the curve (AUC) is represented to the right. (B) Insulin tolerance test (ITT) performed in the same mice of Figure 7B and 7C of the main paper. (0.26 MB TIF) [file pone.0008744.s004.tif]

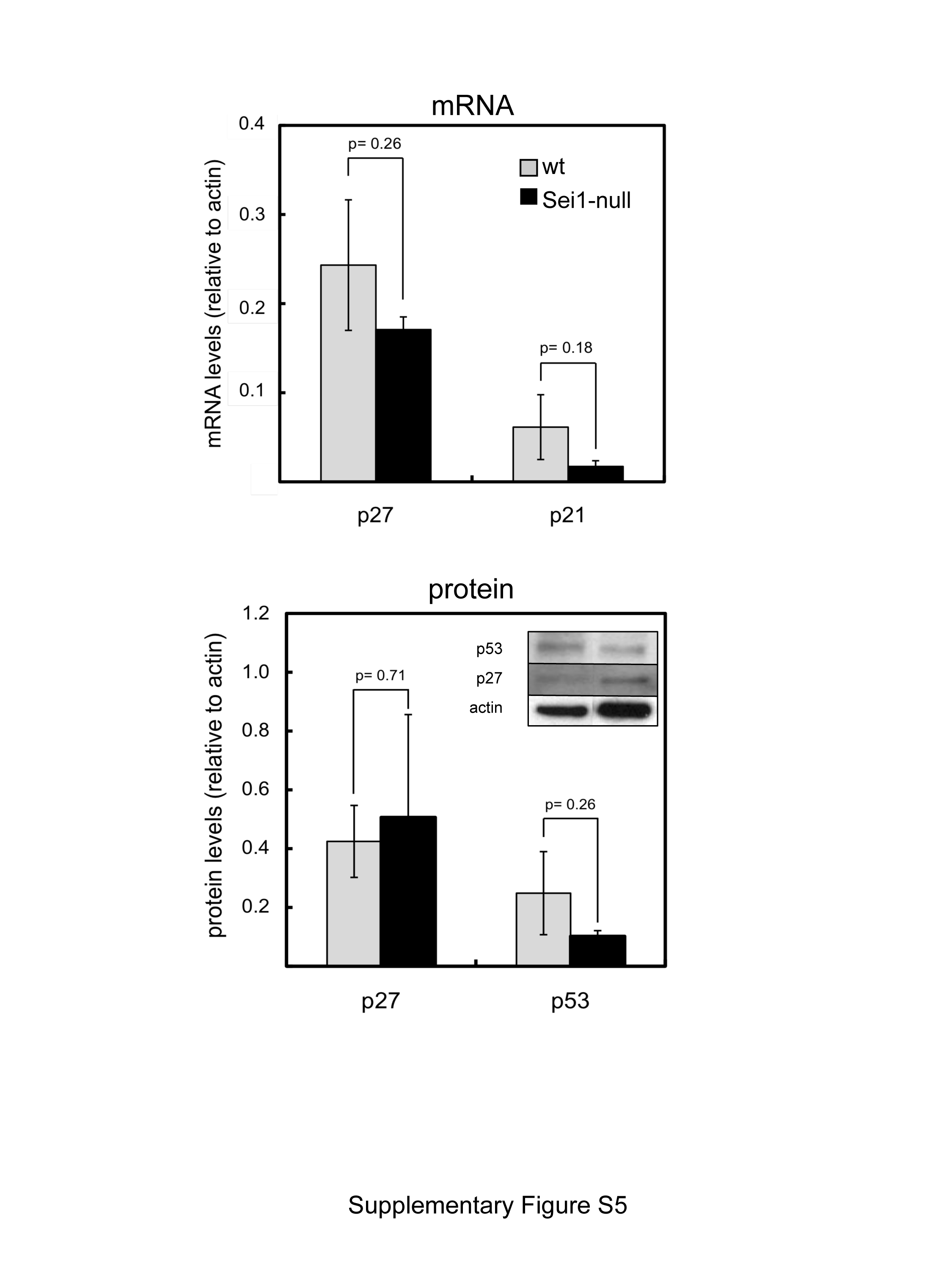

Supplement: Figure S5 — Levels of cell cycle regulators in Sei1-null pancreatic islets. Islets were isolated from wt and Sei1-null mice (n = 3 for each genotype) and each individual preparation was analyzed separately. RNA levels were measured by quantitative real-time PCR and protein levels by immunoblot (the inset shows one example per genotype). Student's t-test was used to compare the two genotypes but no significant differences were found. (0.32 MB TIF) [file pone.0008744.s005.tif]
